# Crystal Structures of Human Muscle Fructose-1,6-Bisphosphatase: Novel Quaternary States, Enhanced AMP Affinity, and Allosteric Signal Transmission Pathway

**DOI:** 10.1371/journal.pone.0071242

**Published:** 2013-09-27

**Authors:** Rong Shi, Ze-Yong Chen, Dao-Wei Zhu, Chunmin Li, Yufei Shan, Genjun Xu, Sheng-Xiang Lin

**Affiliations:** 1 Laboratory of Molecular Endocrinology and Oncology, Centre Hospitalier Université de Québec Research Center (CHUQ-CHUL), Department of Molecular Medicine and PROTEO, Laval University, Québec City, Canada; 2 Département de Biochimie, de Microbiologie et de Bio-Informatique, IBIS et PROTEO, Université Laval, Pavillon Charles-Eugène Marchand, Québec City, Canada; 3 Institute of Biochemistry and Cell Biology, Shanghai Institutes for Biological Sciences, Shanghai, China; 4 The Laboratory of Structural Biology for Visiting Scientists at Institute of Biochemistry and Cell Biology, Shanghai Institutes for Biological Sciences, Shanghai, China; Griffith University, Australia

## Abstract

Fructose-1,6-bisphosphatase, a key enzyme in gluconeogenesis, is subject to metabolic regulation. The human muscle isozyme is significantly more sensitive towards the allosteric inhibitor, AMP, than the liver isoform. Here we report crystal structures and kinetic studies for wild-type human muscle Fru-1,6-Pase, the AMP-bound (1.6 Å), and product-bound complexes of the Q32R mutant, which was firstly introduced by an error in the cloning. Our high-resolution structure reveals for the first time that the higher sensitivity of the muscle isozyme towards AMP originates from an additional water-mediated, H-bonded network established between AMP and the binding pocket. Also present in our structures are a metaphosphate molecule, alternate conformations of Glu97 coordinating Mg^2+^, and possible metal migration during catalysis. Although the individual subunit is similar to previously reported Fru-1,6-Pase structures, the tetrameric assembly of all these structures deviates from the canonical R- or T-states, representing novel tetrameric assemblies. Intriguingly, the concentration of AMP required for 50% inhibition of the Q32R mutant is increased 19-fold, and the cooperativity of both AMP and Mg^2+^ is abolished or decreased. These structures demonstrate the Q32R mutation affects the conformations of both N-terminal residues and the dynamic loop 52–72. Also importantly, structural comparison indicates that this mutation in helix α2 is detrimental to the R-to-T conversion as evidenced by the absence of quaternary structural changes upon AMP binding, providing direct evidence for the critical role of helix α2 in the allosteric signal transduction.

## Introduction

Fructose-1,6-bisphosphatase (Fru-1,6-Pase) catalyzes the hydrolysis of fructose-1,6-bisphosphate (F1,6P_2_) to fructose6-phosphate (F6P) and inorganic phosphate [1]. The enzyme is a primary control point in gluconeogenesis and is important for the regulation of blood glucose [2]. Fru-1,6-Pase is regulated by two inhibitors: AMP, which binds at an allosteric site, and fructose-2,6-bisphosphate (F2,6P_2_), which binds to the active site. These two inhibitors act synergistically to regulate Fru-1,6-Pase activity [2], [3]. The enzyme requires divalent cations (Mg^2+^, Mn^2+^, and/or Zn^2+^) for activity, and monovalent cations such as K^+^, NH_4_^+^, Tl^+^ to further enhance catalysis [3].

In mammals, Fru-1,6-Pase is a homotetramer with a subunit mass of 37,000 Da. Each subunit of the tetramer (designated C1, C2, C3 and C4) has an allosteric AMP domain (residues 1–200) and a catalytic F1,6P_2_ domain (residues 201–335). Extensive structural and functional studies have been carried out on this important enzyme during the last two decades. Crystal structures of mammalian Fru-1,6-Pase from various sources (pig kidney/liver, human liver, and rabbit liver) have been reported [1]–[4]. These X-ray crystallographic studies revealed that Fru-1,6-Pase exists in distinct quaternary conformations (T-state and R-state), depending on the ligands bound to the protein. In the absence of AMP, Fru-1,6-Pase is in its active R-state, in the presence or absence of metal cations and/or other active site ligands. In the presence of AMP, the top pair of subunits rotates 15∼17° relative to the bottom pair, resulting in the inactive T-state conformer. In recent years, the tetramer assembly of Fru-1,6-Pases from different sources (*e.g.*, pig kidney, and more distantly *Escherichia coli*), has been shown to be more complicated than described above. Several different quaternary conformations have been reported [5]–[9]. Various crystal structures have also provided important insight into the mechanisms of catalysis and inhibition of Fru-1,6-Pase [10], [11]. In parallel with crystallographic studies, a series of enzyme kinetic studies on the various mutants revealed the roles of many residues in different regions of Fru-1,6-Pases, and these results have significantly improved our understanding of the enzyme [2], [12]–[16].

Previous studies indicated that the *K*i values for inhibition by AMP vary: <1 µM for the rabbit skeletal muscle enzyme [17], 10–20 µM for the liver and kidney enzymes [18], 80–200 µM for the yeast enzymes [19], [20], and no inhibition for the chloroplast and bumblebee flight muscle enzymes [21]. Despite strong similarity among the nucleotide binding domains, the mammalian muscle Fru-1,6-Pase can be distinguished from the same enzyme from other sources by several kinetic properties, *e.g.*, the higher affinity (10–100 times lower *I*_0.5_, *i.e.*, the inhibitor concentration which causes 50% reduction of the enzyme catalysis) for AMP than either the liver or kidney enzymes [22]–[26]. Recent kinetic studies on mutant forms of human muscle and liver Fru-1,6-Pase indicated that Lys20 plays a pivotal role in the high affinity of human muscle Fru-1,6-Pase (hmFru-1,6-Pase) for AMP [25], [26]. A comparison of the homology between Fru-1,6-pase from human muscle and human liver revealed that they share 77% sequence identity, and that muscle Fru-1,6-Pase must be encoded by a separate gene [23]. It was also suggested that the different Fru-1,6-Pase proteins may have evolved according to the specific metabolic needs of different tissues. In addition to being a specific glyconeogenic enzyme in muscle, Fru-1,6-Pase was proposed to have an additional role in both heat production contributing to the maintenance of body temperature and amplification of flux regulation of glycolysis versus glyconeogenesis [27], [28]. In gluconeogenesis, as well as in glyconeogenesis, the substrate for muscle Fru-1,6-Pase is supplied by aldolase. Muscle aldolase strongly interacts with muscle Fru-1,6-Pase resulting in the formation of a heterologous Fru-1,6-Pase-aldolase complex, which is not sensitive to AMP inhibition [29].

We previously reported the crystallization of hmFru-1,6-Pase [30]. Thanks to the high resolution of the structure, we realized during the structure determination step that one error was introduced in the cloning step resulting in the mutation Q32R. Since the mutant displayed interesting structural and kinetic properties, we then re-cloned the wild-type gene followed by structure-function studies. During the preparation of this manuscript, a paper describing the AMP-bound T-state structure of the hmFru-1,6-Pase E69Q mutant was published, in which a T-state structure, similar to other AMP-bound Fru-1,6-Pases, was described [31]. However, the mechanism underlying higher sensitivity towards AMP was not addressed in this recent paper. Here we report the crystal structures of the wild-type hmFru-1,6-Pase, and the product and AMP complexes of its Q32R mutant. Interestingly, these structures demonstrate novel tetrameric assemblies. More importantly, the 1.6 Å AMP-bound structure reveals for the first time the molecular basis for the enhanced sensitivity of hmFru-1,6-Pase to AMP. This structure also constitutes the first example that AMP binding fails to induce any further tetrameric conformational changes in Fru-1,6-Pase. These structures, together with kinetic studies of the Q32R enzyme, reveal how a mutation in the helix α2 can decrease the sensitivity towards AMP, and eliminate the cooperativity of both AMP and Mg^2+^.

## Materials and Methods

### Expression, purification and crystallization

The wild-type and Q32R hmFru-1,6-Pase enzymes were overproduced in *Escherichia coli* and prepared as previously described [23], [30]. The wild-type protein was crystallized by the vapor-diffusion technique in hanging drops [30]. The protein sample was concentrated to 15 mg/ml in a buffer containing 50 mM Hepes pH 7.0, 0.4 mM EDTA, 0.4 mM dithiothreitol (DTT), 100 mM KCl, 10% (v/v) butanediol, and 0.06% (w/v) β-octylglucoside. Crystals were obtained by mixing equal volumes of the protein sample and a reservoir solution containing 100 mM MgCl_2_, 15% PEG-4000, and 100 mM Hepes pH 7.5 at 23°C. 5 mM F-6-P and phosphate, or 2 mM AMP were added to the Q32R mutant to obtain product or inhibitor complexes. These crystals all belonged to space group *P*4_2_2_1_2 with a = 73.9, c = 146.7 Å, and contained one monomer in the asymmetric unit with a Vm of 2.70 Å^3^ Da^−1^, corresponding to a solvent content of 54.0%. Crystals were transferred to reservoir solution supplemented with 12% (v/v) ethylene glycol, and flash cooled in a nitrogen stream at 100 K (Oxford Cryosystems) for data collection.

### Kinetic studies and Hill coefficient determination

Kinetic measurements were carried out in a reaction mixture containing 25 mM Hepes pH 7.5, 150 mM KCl, 200 µM NADP, 50 µM F-1,6-P_2_, and the coupling enzyme glucose-6-phosphate dehydrogenase/phosphoglucose isomerase at a ratio of 1∶200 (10 units/ml). Human Fru-1,6-Pase was added to a final concentration of 50 µg/ml in a 400 µl reaction mixture for the kinetic measurements. The absorption variation at 340 nm was measured as a function of time. The *k*_cat_ and *K*_m_ values were obtained by varying F-1,6-P_2_ from 0.1 to 100 µM at a saturating concentration of Mg^2+^. One unit of enzyme is defined as the quantity of hmFru-1,6-Pase, which converts 1 µmole substrate into product at 25°C and pH 7.4 corresponding to the maximum velocity obtained with saturating F-1,6-P_2_ and Mg^2+^. The Mg^2+^ ion concentration was varied from 1.0 to 4.5 mM for the determination of the Hill coefficient for Mg^2+^. 4.5 mM MgCl_2_ was included in the reaction mixture for determination of the Hill coefficients for AMP. The initial velocity was followed by increasing the concentration of AMP from 0, 0.1 to 8 µM.

The following equation was used to plot the Hill coefficient of Mg^2+^,
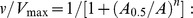
where A is the Mg^2+^ concentration in mM, *A*_0.5_ the Mg^2+^ concentration that yields a *v* = 0.5 *V*_max_, *n* the Hill coefficient, *V*_max_ the maximal velocity that can be obtained at saturating F-1,6-P_2_ and saturating Mg^2+^ concentrations. Similarly, the equation
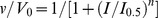
was used to plot the Hill coefficient of AMP, in which *I* indicates the concentration of AMP in µM, *I*_0.5_ the concentration of AMP when *v* = 0.5*V*_0_, and *V*_0_ is the velocity in the absence of AMP.

### Data collection, structure determination and refinement

The data set for the wild-type hmFru-1,6-Pase crystals was collected to 2.69 Å using the R-axis-IIc image plate detector on a Rigaku RU-200 rotating anode at our home facility. Data collection for the Q32R mutant in complex with AMP was performed at the X8C beamline, NSLS at Brookhaven National Laboratory, New York, USA. Complete data were collected to 1.6 Å resolution. Data for the Q32R mutant in complex with products (F6P+Pi) were collected to 2.23 Å at the beamlines 31-LRL-CAT and 19BM at the Advanced Photon Source (APS), Argonne National Laboratory, Chicago, USA. All diffraction data were processed using the HKL program suite [32]. Structure determination was performed by molecular replacement using the program Molrep [33] from the CCP4 suite with the porcine kidney Fru-1,6-Pase structure [10] (PDB entry codes: 1EYI and 1EYJ), omitting solvent molecules and ligands, as the search model. Manual rebuilding of the models was performed in Coot [34], and refinement was carried out with the Refmac5 program [35] with final *R*_work_/*R*_free_ values of 0.200/0.252, 0.193/0.212, and 0.181/0.194 for the wild-type hmFru-1,6-Pase, the Q32R mutant product complex, and the Q32R-AMP complex, respectively. The models have good geometry as analyzed by MolProbity [36]. Final data collection and refinement statistics are shown in Table 1. Atomic coordinates have been deposited with the RCSB Protein Data Bank with accession codes 4HE0, 4HE1, and 4HE2.

**Table 1:** AMP influence on the kinetic properties of wild-type and the Q32R mutant of Fru-1,6-Pase.

Kinetic Constants	Wild-type Fru-1,6-Pase	Q32R mutant
*V*_0_ (%)	95.6+/−5.1	100.1+/−4.2
*I*_0.5_ (µM)	0.19+/−0.03	3.65+/−0.77
Hill coefficient AMP	1.33+/−0.19	0.59+/−0.09

## Results

### Kinetic study of wild-type hmFru-1,6-Pase and the Q32R mutant

**Figure 1:**
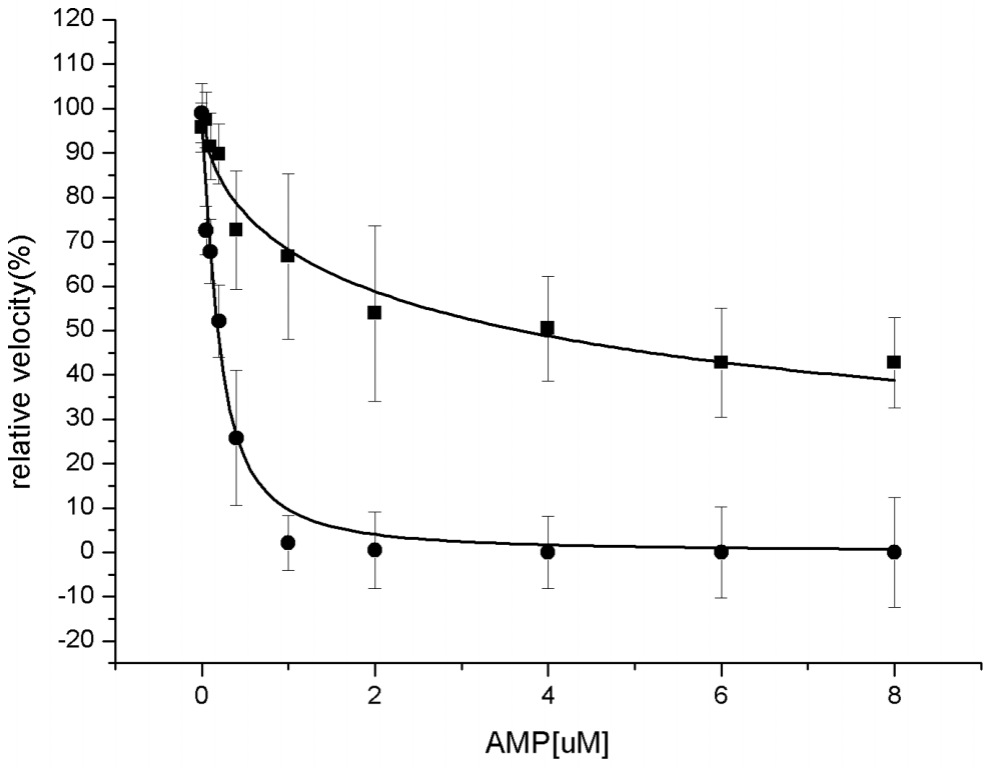
Kinetic properties of wild-type and Q32R mutant of Fru-1,6-Pase. AMP influence on the kinetic properties of Fru-1,6-Pase. Relative (initial) velocity to AMP concentration plot is in full circles for wild type enzyme while full squares for Q32R mutant. Initial velocity in the absence of AMP is taken as 100%. Error bars were for ten experiments.

The *I*_0.5_ value in the study of AMP inhibition, changed from 0.19±0.03 µM to 3.65±0.77 µM for the wild-type hmFru-1,6-Pase and Q32R mutant respectively, showing ∼19-fold modification (Figure 1; Table 1); In parallel, the Hill coefficient decreased less significantly from 1.34±0.19 to 0.59±0.09 showing a 2.3-fold modification.

A similar tendency to the *I*_0.5_ modification for AMP was observed for Mg^2+^: the *A*_0.5_ for Mg^2+^ increased 2.4-fold from the wild-type to the enzyme mutant, while the Hill coefficient decreased 2.1-fold following the variation.

### Structure of the wild-type hmFru-1,6-Pase

**Figure 2:**
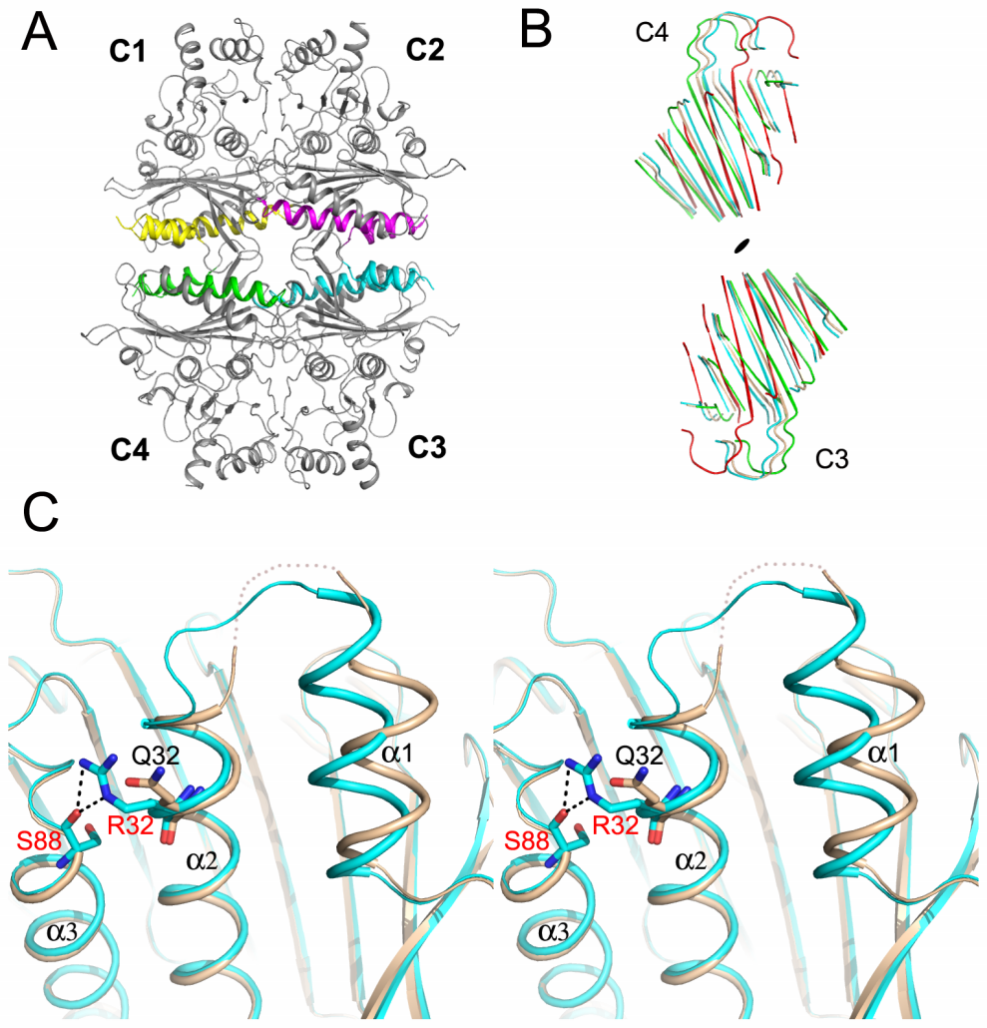
Crystal structures of hmFru-1,6-Pase. A. Cartoon representation of hmFru-1,6-Pase tetramer (side view). The four subunits are designated C1, C2, C3, and C4 with the helices α1 and α2 in these subunits colored in yellow, magenta, cyan, and green, respectively. B. Quaternary states of hmFru-1,6-Pases (the wild-type hmFru-1,6-Pase colored in wheat and the Q32R hmFru-1,6-Pase colored in cyan) relative to the canonical R-state (colored green, PDB code 1CNQ) and the canonical T-state (colored red, PDB code 1EYK). These various structures show remarkable displacement in the Cα atoms of the C3–C4 dimer relative to the C1–C2 dimer. For clarity, only the β-strands of the AMP domain of the C3–C4 dimer are shown here. The superposition of the C1–C2 dimer is based on the established residue subsets [9]. C. Stereoview of the superpostion of the α1 and α2 helices in the wild-type (colored in wheat) and the Q32R mutant (colored in cyan) of hmFru-1,6-Pase.

The hmFru-1,6-Pase crystals in this report are in space group *P*4_2_2_1_2 (X-ray data collection and refinement statistics for all structures are shown in Table 2), which is different from all the previously reported structures of Fru-1,6-Pases, including the AMP-bound T-state hmFru-1,6-Pase that was recently reported [31]. The crystals contain one subunit in each asymmetric unit. The tetramer (Figure 2a) is generated through symmetry operation. The wild-type structure includes residues 7–23 and 27–335. Several residues at the termini, in addition to residues 24–26 could not be traced in the electron density maps. The overall structure is similar to that of other Fru-1,6-Pases as evidenced by an rmsd of 0.60 Å for the 324 Cα atoms when compared with the R-state structure (PDB 1EYI) of porcine Fru-1,6-Pase. The dynamic loop 52–72 adopts the engaged conformation as described for other R-state Fru-1,6-Pases, but the electron density is weaker than other parts of the protein indicating that this loop is more mobile. The active site contains three metal ions (designated M1, M2, and M3 as previously described [10] and one phosphate molecule. The temperature factor of M3 (57 Å^2^) is significantly higher than that of M1 and M2 (∼34 Å^2^ for both), and is possibly a result of fewer coordinating ligands (two compared to the 5 or 6 coordinations of M1 and M2), or it may simply be a water molecule. It is notable that the location of M1 is ∼1 Å from M1 in the R-state porcine Fru-1,6-Pase (*e.g.*, PDB 1EYI, 1CNQ, 1NUY). Superposition of the wild-type hmFru-1,6-Pase tetramer onto the canonical R-state tetramer using the established residue subsets [9] reveals a change in the tetramer assembly (Figure 2b). The top pair C1–C2 underwent a 3° rotation relative to the bottom pair, C3–C4, when compared with the canonical R-state, being 12° away from the canonical T-state.

**Table 2:** X-ray data collection and refinement statistics.

Structure	Q32-hmFru-1,6-Pase (Pi+Mg^2+^)	R32-hmFru-1,6-Pase (F-6-P+Pi+Mg^2+^)	R32-hmFru-1,6-Pase (AMP+PO_3_+Mg^2+^)
Space group	P4_2_2_1_2	P4_2_2_1_2	P4_2_2_1_2
*a, c* (Å)	73.9, 146.7	73.8, 146.6	73.4, 146.6
wavelength (Å)	1.5418	0.9000	0.9795
resolution^a^ (Å)	50–2.69 (2.79–2.69)	50–2.23 (2.31–2.23)	50–1.60 (1.66–1.60)
observed *hkl*	105347	279468	347718
unique *hkl*	11946	20427	51809
redundancy	8.8	13.7	6.7
completeness (%)	99.9 (100)	99.5 (100)	96.5 (98.7)
R*_sym_* b	0.184 (0.509)	0.104 (0.500)	0.051 (0.493)
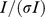	13.6 (3.2)	25.1 (5.7)	34.6 (4.2)
Wilson B (Å^2^)	51.5	33.1	21.0
R*_work_* c (# *hkl*)	0.199 (11341)	0.193 (19339)	0.181 (49142)
R*_free_* (# *hkl*)	0.253 (568)	0.212 (1045)	0.194 (2642)
B-factors (# atoms)			
protein	35.2 (2489)	31.8 (2367)	21.7 (2419)
solvent	18.9 (52)	30.5 (134)	28.7 (257)
ligands	49.4 (9)	40.3 (24)	26.4 (32)
Ramachandran			
favoured (%)	96.9	98.4	99.1
generous (%)	3.1	1.6	0.9
disallowed (%)	0	0	0
rmsd's			
bonds (Å)	0.012	0.010	0.009
angles (°)	1.39	1.21	1.27
*PDB code*	4HE0	4HE1	4HE2

a Values in parentheses (except those corresponding to the number of atoms in the case of the B-factors) represent data from the highest resolution shell.

b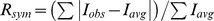.

c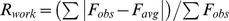.

### Product complex of the Q32R mutant

To obtain structural evidence for the role of α1 and α2 helices in allosteric regulation, we determined the crystal structures of the hmFru-1,6-Pase Q32R mutant. The crystals of the product complex of Q32R hmFru-1,6-Pase are isomorphous with the wild-type crystals. The model includes residues 10–54 and 72–335. Comparison of this model with the wild-type structure yields an rmsd of 0.53 Å for the 304 Cα atoms.

In the Q32R mutant structure, there was no visible electron density for the residues 55–71 indicating that these residues were not ordered in the crystal. However, it should be noted that the conformation of the hinge (residues 51–54) in the Q32R mutant is the same as that in the engaged conformation for wild-type hmFru-1,6-Pase, but different from the disengaged conformation observed for the T-state hmFru-1,6-Pase [31] (PDB 3IFA). Other than this difference, superposition of the wild-type and Q32R mutant structures revealed that this variation induces significant conformational adjustments of the α1 and α2 helices (Figure 2c). Replacement of the glutamine by an arginine residue creates hydrogen bonds between the side chain of Arg32 and the carbonyl group of Ser88, which helps to pull the Cα atoms of residues 28–32 between 0.6–1.5 Å away from the center of the tetramer. This change consequently propagates across the region linking the α1 and α2 helices to translocate the α1 helix of the Cα atoms of residues 14–20 by an average of 2.2 Å toward the center of tetramer. The relative movement of α1 and α2 is reminiscent of the AMP binding event although no AMP is present in the current structure. These conformational changes subsequently affect the residues at the N-terminal. For example, the side chain of Arg15, which forms a salt bridge interaction with Asp9 in the wild-type protein, was disordered in the Q32R mutant. The loss of the stabilizing interaction by Arg15 thus releases Asp9 from its original position and as a result, the well-defined Glu7–Asp9 residues in the wild-type structure become disordered.

Furthermore, inspection of the quaternary state indicates that, compared with the wild-type structure, the C1–C2 pair in the mutant structure has undergone a further rotation by an angle of 2° relative to the C3–C4 pair (Figure 2b). Thus, the tetramer is in an intermediate quaternary state which is 5° away from the canonical R-state and 10° toward the canonical T-state.

One F6P, one phosphate, and two Mg^2+^ ions (M1 and M2) are found in the active site. Based on the electron density maps, the F6P molecule is present as the β-anomer, which differs from the α-anomer observed in the recently published T-state hmFru-1,6-Pase structure [31]. Notably, in accord with the slight movement of the side chain of Asp121, M1 is located 0.7 Å away from M1 in the wild-type structure, and is 1.7 Å distant from M1 in the product complexes of porcine Fru-1,6-Pase (PDB codes 1EYI and 1CNQ). Moreover, Glu97 adopts a different conformation compared with that observed in the above structures. The relative positioning of Asp121, Glu97, and M1 is almost identical to that observed in the high pH product complex of porcine Fru-1,6-Pase (PDB 1NUW) although the current Q32R mutant structure was obtained from crystals grown at neutral pH. Notable conformational changes also include Arg276, which now swings out towards the protein surface and would undergo steric clashes with Thr66 if the loop 52–72 adopted an engaged conformation.

### AMP complex of the Q32R mutant

The structure model of the AMP complex includes residues 11–54 and 72–335. The dynamic loop remains disordered. Unexpectedly, binding of AMP does not lead to significant conformational changes either at the monomer level, or at the quaternary level compared with the product complex of the Q32R mutant. The rmsd between these two structures is 0.29 Å for the 308 Cα atoms. Only minor changes occur at the AMP binding site, for example, the α1 helix shifts ∼0.4 Å to accommodate the AMP molecule.

**Figure 3:**
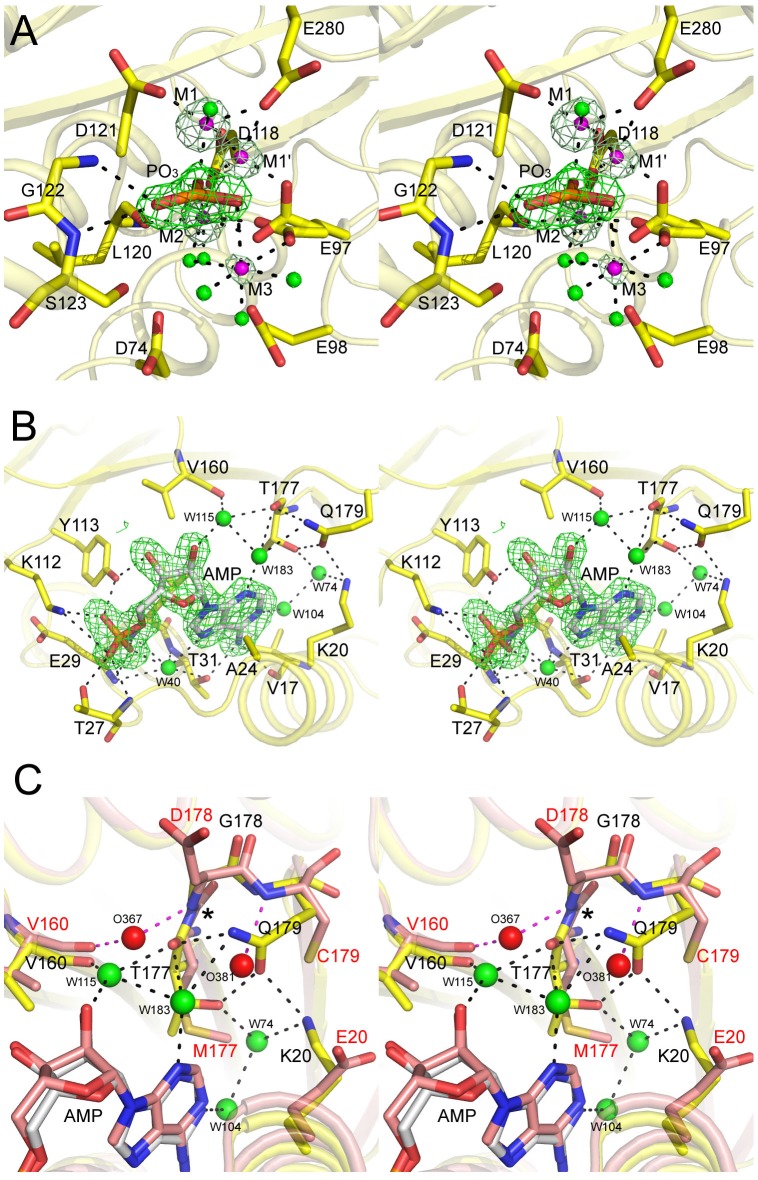
Stereoviews of the AMP complex of the Q32R hmFru-1,6-Pase. The metal ions and water molecules are represented by magenta and green spheres, respectively in hmFru-1,6-Pase. The carbon atoms for the hmFru-1,6-Pase and AMP are in yellow and white, respectively. A. The presence of a metaphosphate (PO_3_) and metal ions (labeled M1, M1′, M2 and M3) in the active site. The Fourier difference maps with the above atoms omitted for calculation cover the metaphosphate molecule and metal ions at a contour level of 6σ. B. Interactions between AMP and the binding pocket. The Fourier difference map for AMP contoured at 2.5σ is shown as a green mesh. Direct and water-mediated H-bonds are depicted by black dash lines. The water molecules contributing to AMP binding are labeled: the water W40 connecting the phosphate moiety and the adenine moiety is commonly observed in hmFru-1,6-Pase and other Fru-1,6-Pases, whereas W115 and W183 are unique to hmFru-1,6-Pase. C. Superposition of the AMP binding pocket of hmFru-1,6-Pase (residues labeled in black), and human liver Fru-1,6-Pase (PDB 1FTA)(residues labeled in red). The carbon atoms for the human liver Fru-1,6-Pase are colored salmon, and the two water molecules (O367 and O381) bound in the pocket (albeit too far to form H-bonds to AMP) are represented by red spheres and their interactions with human liver Fru-1,6-Pase are shown as magenta dashed lines. The flip of the peptidyl bond connecting residues 177 and 178 resulting from sequence variation between hmFru-1,6-Pase and human liver Fru-1,6-Pase in this region is indicated by the black asterix.

The electron density maps clearly support the presence of a metaphosphate molecule at the active site (Figure 3a) since the phosphoryl atom and the other three oxygen atoms are nearly in the same plane, and refinement with orthophosphate does not provide a good fit. Observation of metaphosphate in this location has previously been reported [11]. The active site of a small percentage of Fru-1,6-Pase molecules in the crystal may be occupied by the F6P molecule, but the electron density is very weak. The active site also contains four metal ions: M1, M1′, M2, and M3 as can be seen from their strong electron density at high contour level (Figure 3a). The stabilization of metaphosphate mainly relies on interactions with these metal ions and the hydrogen bonds with the backbone amides of Gly122 and Ser123. The salt bridge interaction between the metaphosphate and the side chain of Arg276, as shown in the previous metaphosphate complexes, is not present because Arg276 remains pointed towards the protein surface.

M1 corresponds to the metal ion M1 in the product complexes of porcine Fru-1,6-Pase, and has five inner-sphere ligands: one oxygen from each of Asp118, Asp121, Glu280, metaphosphate, and water 270. M1′ is an alternate M1 and corresponds to the metal ion M1 in the product complex of the Q32R mutant as described above. It has 4 coordinating oxygen atoms from Glu97, Asp118, Glu280, and metaphosphate. Both M2 and M3 are six-coordinated: M2 is stabilized by Glu97, Asp118, Leu120 (carbonyl), metaphosphate, and two water molecules (W28 and W67), whereas M3 is coordinated by Glu97, metaphosphate, and four water molecules (W67, W110, W143, and W289). M1 and M1′ are likely exclusive as they are only 1.7 Å apart. To resolve this problem and obtain compatible temperature factors for the ligands relative to the coordinating protein atoms, the occupancies of M1 and M1′ were set to 0.75 and 0.25, respectively. In addition, the occupancy of M3 was set to 0.50, under which there was no negative or positive density in the difference maps. For the same reason, the occupancy of metaphosphate was also set to 0.75. The resulting temperature factors of these ligands were comparable with the surrounding atoms, and no negative or positive densities were presented at these positions in the Fourier difference map. Unexpectedly, the side chain of Glu97 has alternate conformations: in one conformer, the carboxylate oxygens coordinate M1′ and M2, whereas in the second conformer, they bridge M2 and M3. It should be pointed out that M1′ is not likely to be Mg^2+^, but may be a Zn^2+^ cation. When modeling was carried out with Mg^2+^ at an occupancy of 0.25, the refinement yielded a considerably lower temperature factor (6.3 Å^2^) compared to that of the surrounding residues (∼17 Å^2^), and some positive electron density remained at this site. In contrast, modeling Zn^2+^ at this position with 0.25 occupancy resulted in a compatible temperature factor (17.2 Å^2^). Meanwhile, the positive peaks disappeared on the Fourier difference maps. The presence of a Zn^2+^ ion at this position is also supported by its decreasing coordination numbers.

The present structure (1.6 Å) has the highest resolution among all published AMP-bound Fru-1,6-Pases. AMP is well defined due to its strong electron density (Figure 3b). Superposition of the current structure with the structures of porcine or human liver Fru-1,6-Pase [2] reveals an almost identical binding mode for AMP at the allosteric site: it is stabilized by hydrogen bonding interactions with residues Val17, Thr27, Glu29, Leu30, Thr31, Lys112, and Tyr113. Meanwhile, the adenine ring is sandwiched between Gly20 and Ala24 on one side, and Leu30 (or Met30) on the other side. The sequence differences between the AMP binding site in human liver and muscle Fru-1,6-Pases are mainly restricted to two locations: Glu20 in human liver Fru-1,6-Pase is replaced by Lys20 in hmFru-1,6-Pase, and residues 176-AMDC-179 are changed to 176-STGQ-179. The latter connects two anti-parallel beta-strands β6 and β7, and is the longest segment showing significant sequence variation between these two isozymes. Of particular interest is that the sequence difference in this segment leads to remarkable conformational deviations in these two isozymes: the peptide bond linking residues 177 and 178 has been flipped (Figure 3c). As a result, the carbonyl group of Thr177 in hmFru-1,6-Pase points directly toward the AMP molecule. This is in contrast to human liver Fru-1,6-Pase where the carbonyl group of Met177 points toward the amide group of Gly180, and is involved in the main-chain hydrogen bonding interactions between the two beta-strands. Interestingly, this structural variation results in a four-residue beta-turn (177-TGQG-180) in hmFru-1,6-Pase in contrast to a two-residue beta-turn 178-DC-179 in human liver Fru-1,6-Pase. Most importantly, such an arrangement in hmFru-1,6-Pase leads to at least two additional water-mediated hydrogen bonding interactions with AMP (Figure 3b): one water (W115) coordinated by the carbonyl groups of both Val160 and Thr177 hydrogen bonds to the ribose hydroxyl (O2′) group of AMP, and the second water (W183) anchored by the carbonyl of Thr177 and the side chain amide (carboxamide) of Gln179 makes a hydrogen bond to the N3 atom of the AMP adenine ring. These two water molecules are 3.1 Å apart. This water-mediated H-bonding network is strengthened by the relative positioning of Thr177, Gln179, and Lys20: the side chain amide group of Gln179 is within hydrogen bond distance of the main chain carbonyl of Thr177, and its side chain carbonyl (carboxamide) is further stabilized by the hydroxyl of Thr177 and the side chain of Lys20 (Figure 3b). These interactions fix the side chain of Gln179, which is located on the protein surface. In the human or porcine liver Fru-1,6-Pases, the water (*e.g.*, O367 in Figure 3, which is 1.9 Å from W115 described above) coordinated by the Val160 carbonyl group and the amide group of Met177 is too far (∼4.5 Å) to interact with the ribose hydroxyl group of AMP (Figure 3c). Furthermore, the second water molecule does not exist even in similar high-resolution structures (*e.g.*, 1YYZ and 2F3D) of mammalian Fru-1,6-Pases.

## Discussion

### Novel tetramer assemblies of mammalian Fru-1,6-Pases

We have presented two novel tetramer assemblies of hmFru-1,6-Pase (Figure 2b), one for wild-type hmFru-1,6-Pase rotated 3° from the canonical R-state, and another for the Q32R mutant rotated 5° away from the canonical R-state. Although the wild-type hmFru-1,6-Pase has a unique quaternary state in the absence of AMP (3° rotation relative to the canonical R-state), it adopts an engaged loop with magnesium ions bound to the active site, likely representing the real R-state of hmFru-1,6-Pase. Therefore, the R-state may not be conserved as previously suggested [37]. Since the AMP-bound hmFru-1,6-Pase (16° rotation relative to the canonical R-state) is a canonical T-state [31], it is very likely that hmFru-1,6-Pase undergoes a rotation of only 13° during the R-to-T transition. Studies on Fru-1,6-Pase from spinach chloroplast showed that the corresponding rotation is 20° (5° relative to the canonical T-state), defining a “super T” state which is insensitive to AMP inhibition [38]. Of interest is that hmFru-1,6-Pase, which undergoes a smaller rotation during the R-to-T transition, is more sensitive to AMP inhibition than its liver counterparts. The current results and previous studies (summarized in [8]) indicate that the Fru-1,6-Pase tetramer assembly in different species, or under different conditions, exhibits great variations (0°, 3°, 5°, 6°, 9°, 12°, 13°, 15°, 16°, and 20° relative to the canonical R-state) and may accommodate more conformations than currently known.

### Active site

Although metaphosphate has been found in the Fru-1,6-Pase active site, this observation was made either at alkaline pH, or at a high (200 mM) K^+^ concentration. Capture of reactive metaphosphate in the active site of human muscle Fru-1,6-Pase is achieved under neutral pH and median concentrations of metal ions (100 mM KCl present in protein buffer). This provides good support for the dissociative mechanism for Fru-1,6-Pases as described previously [11]. The alternative conformations of Glu97, and migration of the metal ion M1 (M1 and M1′) in the active site (Figure 3a) strongly suggest the conformational adjustment of the active site in different catalytic steps.

### Mutation Q32R impairs the transition towards the T-state

We have shown that replacing Q32 by an arginine residue caused dramatic conformational changes in the α1 and α2 helices. One outcome is that certain residues at the N-terminal and in the dynamic loop became disordered. Nevertheless, the loop is not in a disengaged conformation as judged by the conformation of hinge residues 51–54. Therefore, the mutant is still active, but the equilibrium may shift from an engaged to disordered state. As previously suggested the N-terminal residues, located between helix α3 and the loop 187–194, may be important for the stabilization of the engaged loop by long-range effects [13]. Previous studies have shown that the dynamic loop 52–72 is critical for efficient binding of Mg^2+^ and the cooperativity of Mg^2+^ in Fru-1,6-Pases [12]. This is consistent with the fact that truncation of the first ten residues in hmFru-1,6-Pase results in a mutant with decreased cooperativity (a reduction to 1.36 from 2.04) of Mg^2+^ and 60-fold increase of *A*_0.5_ [39]. For this reason, it is surprising that a potentially more mobile loop results in a higher *A*_0.5_ for Mg^2+^ (9 µM from 4 µM), and a significantly decreased cooperativity of Mg^2+^ (Hill coefficient drop to ∼1) in the Q32R mutant.

**Figure 4:**
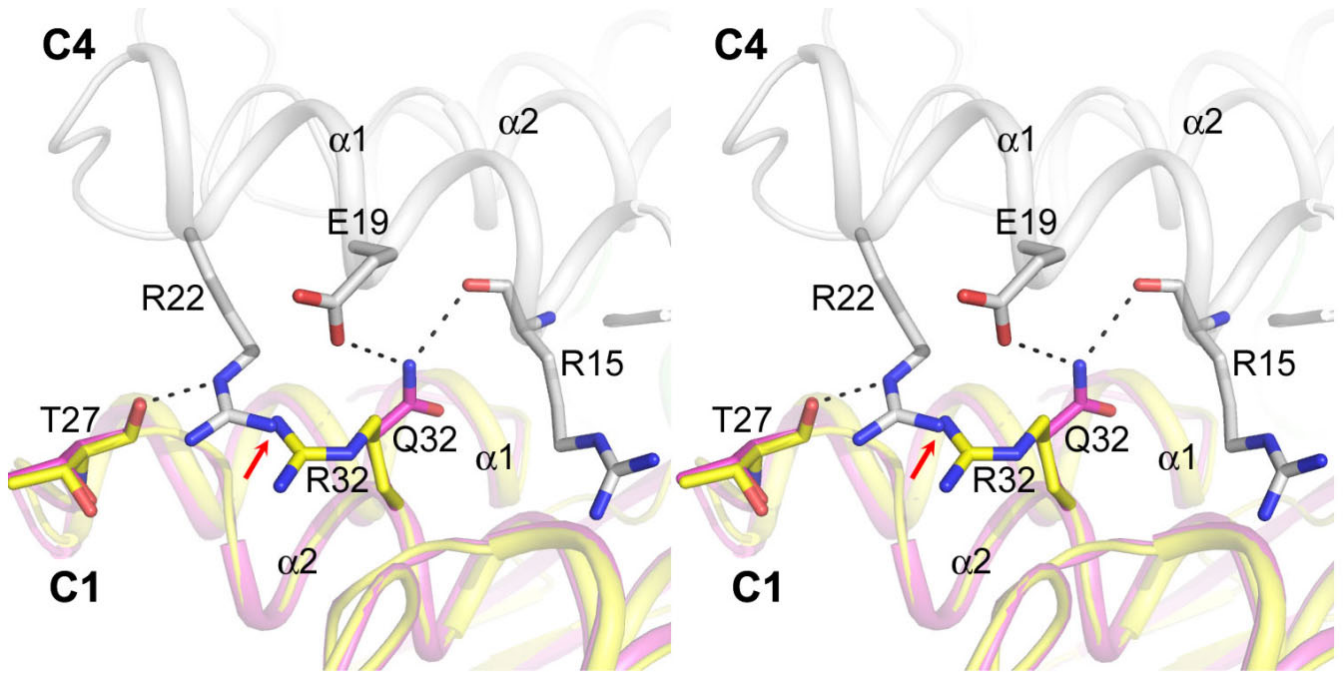
Stereoview of the structure of the Q32R mutant with an unstable T-state. The carbon atoms of the C1 and C4 subunits of the T-state hmFru-1,6-Pase (PDB 3IFA) are colored magenta and white, respectively. The carbon atoms in the Q32R mutant structure (superposed onto the C1 subunit) are colored yellow. The H-bonds between Q32 of the C1 subunit, and R15 and E19 of the C4 subunit are abolished by the Q32R mutation. As a result of potential charge repulsion and steric clashes (indicated by the red arrow), the positively charged bulky side chain of R32 is also detrimental to the formation of the inter-subunit H-bond between T27 in the C1 subunit and R22 in the C4 subunit, a critical interaction for cooperativity.

Probably the most interesting structural observation is that AMP binding does not change the quaternary state of the Q32R hmFru-1,6-Pase as compared with its product complex. This is the first crystallographic observation that AMP binding fails to induce changes in tetramer assembly in any Fru-1,6-Pase. What prevents the transition of the mutant toward the canonical T-state in the presence of AMP? Inspection of the T-state Fru-1,6-Pase structures (PDB codes 1EYJ for porcine liver Fru-1,6-Pase, [10]; 3IFA for hmFru-1,6-Pase, [31]) reveals that in these structures Q32 in subunit C1 hydrogen bonds to the main chain carbonyl group of Arg15 and the carboxyl side chain of Glu19 in subunit C4 (Figure 4). These interactions are important for the stabilization of the T-state. Placing a larger arginine in a similar conformation in this position will inevitably cause steric clashes with the residues in subunit C4. To avoid this, Arg32 has to rotate away; thus, the above-mentioned interactions between C1 and C4 will be lost resulting in the destabilization of the T-state. Moreover, the new position of the Arg32 side chain is incompatible with the conformation of Arg22 (subunit C4) in the T-state due to potential steric hindrance. The latter residue is important for stabilizing the T-state structures (Figure 4). In the AMP-bound T-state structures of hmFru-1,6-Pase [31](PDB codes 3IFA and 3IFC), the NE atom of Arg22 hydrogen binds to the main chain carbonyl group of Thr27 in all subunits. As a result of potential steric clashes and charge repulsion, Arg32 (subunit C1) would efficiently prevent the approach of Arg22 (subunit C4) toward Thr27 (subunit C1) (Figure 4). Therefore, the loss of six hydrogen bonding interactions (Q32-R15′, Q32-E19′, Thr27-Arg22′, R15-Q32′, E19-Q32′, and Arg22-Thr27′) between subunits C1 and C4 as a result of the presence of Arg32 at this position will be detrimental to the conversion to the T-state even with AMP bound to the allosteric site. This explains why the *I*_0.5_ value for AMP increased approximately 19-fold in the mutant compared with the wild-type enzyme. The kinetic measurements also indicated that AMP cooperativity is lost as evidenced by a Hill coefficient of below 1. This is not surprising since the hydrogen bonding interaction between Thr27 (C1) and Arg22 (C4) has been shown to be critical for AMP cooperativity. The latter cooperativity is abolished in the R22M variation [40]. Thus, even the C1 subunit could reach a T-like state: the lack of an Arg22-Thr27 interaction would prevent conveyance of the signal from the AMP binding event in subunit C1 to the opposite subunit C4. It should be noted that it is likely that both the destabilization of the T-state structure, and the lack of Arg22–Thr27 interaction contribute to the loss of cooperativity for AMP. The change in *I*_0.5_ for AMP in the Q32R mutant of hmFru-1,6-Pase shares some similarity with the A54L mutant of pig kidney Fru-1,6-Pase reported previously [9]. In the A54L mutant, the concentration of AMP that causes 50% inhibition increased 50-fold. Interestingly, conformational changes in the α2 and α1 helices were observed in both cases, supporting the notion that these two helices are important for allosteric regulation.

Based on the T-state structures, we could expect that substitution to a less bulky side chain on residue 32 may be detrimental to the stabilization of the T-state, but would not block signal communication between the two subunits C1 and C4. This is consistent with the kinetic results obtained for the Q32L mutant of porcine liver Fru-1,6-Pase [15] in which the Hill coefficient for AMP inhibition is the same as wild-type (2.0), but the *I*_0.5_ increased 8-fold. This is easily understood since the leucine side chain could not fulfill the role of glutamine in forming H-bonds with both Arg15 and Glu19; therefore the presence of a leucine residue would destabilize the T-state complex.

### Structural basis for higher AMP sensitivity in hmFru-1,6-Pase

As previously mentioned, kinetic studies of hmFru-1,6-Pases have demonstrated that they exhibit higher affinity (10–100 times lower I_0.5_) for AMP than either the liver or kidney enzymes. The 1.6 Å AMP complex of hmFru-1,6-Pase reported here provides a structural basis for the underlying mechanism of its higher sensitivity towards AMP. The sequence variation-induced conformational adjustment of the beta-turn β6/β7 created a strong water-mediated H-bonding network to strengthen AMP binding at the allosteric site. This integral network is well maintained by the mutual interactions between Thr177, Gln199, and Lys20 (Figure 3b). Varying any of these residues would destroy this network and decrease its binding affinity for AMP. This is supported by kinetic results from the K20E (22 fold), T177M/Q179C (11 fold), and K20E/T177M/Q179C (26 fold) mutants of human muscle Fru-1,6-Pase showing their decreased sensitivity towards AMP [29]. In fact, the sensitivity of the triple mutant of hmFru-1,6-Pase to AMP is similar to that of human liver Fru-1,6-Pase. On the other hand, human liver Fru-1,6-Pase mutants show increased affinity for TNP-AMP (AMP analog), *e.g.*, the triple mutant E20K/M177T/C179Q of human liver Fru-1,6-Pase has a *K*_D_ (only three times higher) comparable to the wild-type muscle enzyme. Moreover, the affinity of the E20K or M177T/C179Q mutants for TNP-AMP is similar to that of the wild-type liver enzyme. This strongly indicates that changing one (E21K) or two (M177T/C179Q) residues could not fulfill the functional role of the entire H-bonding network established by three residues in the muscle enzyme. However, it should be noted that the increased affinity for AMP in the triple mutant of the human liver enzyme does not lead to a drastic change in *I*_0.5_. It is likely that the pathway for signal transmission has been disturbed in this mutant. Another possibility is that, in addition to its higher affinity, the higher sensitivity towards AMP is the result of a smaller rotation (12∼13° compared with the canonical 15° rotation in liver isozymes) required for the R-to-T transition as discussed above.

In conclusion, the structural and functional studies of wild-type hmFru-1,6-Pase and the Q32R mutant provide novel insights into its enhanced sensitivity for AMP and the signal transmission mechanism during the R-to-T transition. Moreover, the novel tetramer assembly of wild-type hmFru-1,6-Pase indicates that the R-state of Fru-1,6-Pases may not be conserved, and that the muscle enzyme may undergo a smaller rotation in response to AMP inhibition than the enzymes from liver and kidney.
