# Correction: Crystal Structures of Human Muscle Fructose-1,6-Bisphosphatase: Novel Quaternary States, Enhanced AMP Affinity, and Allosteric Signal Transmission Pathway

**DOI:** 10.1371/annotation/6a44f0f6-7013-49bf-b90d-26fc33bdf36e

**Published:** 2014-01-06

**Authors:** Rong Shi, Ze-Yong Chen, Dao-Wei Zhu, Chunmin Li, Yufei Shan, Genjun Xu, Sheng-Xiang Lin

The affiliation of the third author is incorrect. Dao-Wei Zhu is not affiliated with institution number 1, but rather with institution number 2, Laboratory of Molecular Endocrinology and Oncology, Centre Hospitalier Université de Québec Research Center (CHUQ-CHUL), Department of Molecular Medicine and PROTEO, Laval University, Québec City, Canada. 

